# The work experience of a patient affected by Williams Syndrome: a pilot project at the Bambino Gesù Children’s Hospital

**DOI:** 10.1186/s13023-017-0657-6

**Published:** 2017-05-31

**Authors:** Francesca De Lorenzo, Marina Macchiaiolo, Carla Maria Carlevaris, Andrea Bartuli

**Affiliations:** Bambino Gesù Children’s Hospital, Rome, Italy

**Keywords:** Williams Syndrome, Work experience, Inter-individual variability

## Abstract

A new approach has been designed at the Bambino Gesù Children’s Hospital in Rome aimed at increasing empowerment in Williams Syndrome individuals through tutor-assisted work activities. Williams Syndrome is characterized by a combination of distinguishing physical traits, congenital anomalies, intellectual disabilities, and a specific developmental profile.

This manuscript describes the case of a Williams Syndrome patient.

There are only few papers in the scientific literature describing interventions targeting improvement in the quality of life of adult Williams Syndrome individuals. Therefore, this experience may prove useful to several patients, their families, and the experts helping them.

We described an example of intervention aimed at guiding and facilitating a Williams Syndrome patient within a work environment, taking into consideration the peaks and valleys of these individuals’ specific abilities.

Based on our results, we also stressed the need to promote a set of projects and initiatives aimed at enhancing as much as possible self-sufficiency and psycho-affective balance in Williams Syndrome individuals, in order to protect their dignity and self-esteem.

## Introduction

Williams Syndrome (WS) is a rare genetic non-degenerative disorder, with an estimated prevalence of 1 out of 7500/10,000, and with a potentially chronic clinical outcome [15, 18]. It is a multi-systemic condition caused by hemizygous deletion of 1.5 to 1.8 Mb on chromosome 7q11.23, containing approximately 28 genes, that may be involved in executive functioning [14]. This syndrome is characterized by typical dysmorphic features, varying degree of intellectual disabilities, congenital heart defects, and abnormal growth patterns (prenatal and postnatal growth retardation, short stature) [2].

Over the past three decades, scientific research has produced a significant amount of information on the genetic, cognitive and behavioural characteristics of Williams Syndrome patients. Most studies have focused on the analysis of linguistic and visual-spatial skills, with the aim of drawing up a profile that could be generally descriptive of the Syndrome [12, 17].

The review produced by Martens et al. [11] pointed out a limit inherent in research work on Williams Syndrome, due to the very few studies available in relation with the evolutionary trajectories of the cognitive/behavioral profiles. As pointed out by Karmiloff-Smith [9, 10], it is important to consider that the adult phenotype may differ from the child phenotype, because the development trajectories are affected in various ways by the effects of the genetic deletion, with a consequent variability among individuals with WS that cannot be overlooked.

Moreover, research has shown that approximately 40% of all people with intellectual disabilities show marked emotional and behavioural problems that interfere with their daily life [3]. It is therefore crucial, in addition to producing more in-depth scientific knowledge on the Syndrome, to invest on treatment options for an improved quality of life and social functioning in WS patients.

Currently, there is no specific cure for WS, but rehabilitation therapy and early targeted educational interventions are extremely important to enhance the development of cognitive and social competencies. Some programs have been developed in recent years to optimize the life performances of children or adults with disabilities; however, most interventions might be considered as “generic”, as they dedicate little attention to the specific characteristics of the child with intellectual disabilities [5].

The Bambino Gesù Children’s Hospital research group (Department of Rare and Genetic Diseases) has tried to create a network to provide support to WS patients. This has been a starting point to improve these patients’ capabilities and help them build awareness on their limits. The aim was to guide and facilitate patients in their work environment, with the ultimate goal of improving their quality of life.

Empowerment is therefore a process of growth of the individual within his or her context of reference. It is also a social process of recognition, promotion and enhancement of the ability to mobilize the necessary resources to achieve one’s goals, which allows patients to perceive they are in control of their own life [6].

## Objectives of the intervention program

The general goals of this project in relation to the WS patient under our care, were:

- To help our patient develop awareness on her internal and external resources, ability to self-monitor such resources and effectively mobilize them in order to trigger off a positive change;
- to plan and make her carry out activities which seemed adequate to her as a person and not only as a Williams syndrome patient.

More specifically, the project aimed to:

- create the best atmosphere to help her fulfill her potential, while supporting her growth, and developing her self-esteem and sense of identity;
- make the patient experience the fulfillment of her wishes and motivations;
- help her modulate her relational and cooperational skills in order to make them appropriate to the context;
- gradually increase work tasks, through tools and activities, which the patient may find engaging in terms of interest and motivation;
- encourage her active participation in the discussion and monitoring of her performances;
- systematically collect data to monitor the project progress, and the results of the different activities.

## WWH: Who – Where - How

The patient who participated in our study is a 27-year old girl and she possesses adequate cognitive, cultural and social skills. She exhibited cooperation and participated willingly in all the activities throughout the project.

The work activities took place twice a week in two different facilities: the Hospital Playroom (used by both inpatients and outpatients) and the Administration office of the Department of Rare Diseases.

The Hospital Playroom was mainly used for activities such as:

- Managing, organizing and monitoring the materials of the facility.
- Contribute to the organization and creation of games for children who used the playroom.
- Receiving the playroom users to illustrate the facility.

The activities carried out in the Administration office comprised:

- Cataloguing and managing stationery.
- Data entry in the internal database of the Hospital Rare Diseases Department.
- Preparing simple documents/reports on her own activity using Microsoft Office applications (Word and Power Point).

A number of constraints can be identified in the neurobehavioral profile of WS patients. These can interfere with the normal development of the individual and become obstacles when entering the labour market, thus becoming “risk factors”. Such constraints include:

i) a learning deficit and the consequent tendency to be distracted while carrying out more complex tasks. Also in a recent study, WS individuals showed inhibition deficits, problems in re-engaging attentional control processes after making an error, and generalized deficits of concentration [7]. Overall, these findings indicate deficits of specific executive functions in WS. Knowing that WS patients learn more easily by observation and imitation than by trial and error [2], we did not use only verbal description to give her instructions on her tasks (despite her appropriate understanding of language), but we also asked her to observe our behavior and emulate it.
ii) A personality profile characterized by a combination of high sensitivity to criticism and low frustration tolerance.
  In this respect, we observed that, for our patient, carrying out tasks was accompanied by social anxiety, fear of failure, and frustration. Therefore, we designed our intervention as follows: every attempt to carry out a task was followed by a break, during which we had the opportunity to check if the task had been performed correctly and monitor our patient’s emotional reaction (frustration or satisfaction, anger/disapppointment or gratification). This section proved very useful for the enhancement of our patient’s self-awareness: she started to become aware of her actions, of her actual skills and of the potential skills she may acquire with time.
iii) High levels of anxiety when unexpected changes occur.
  Taking this aspect into account, we established that all activities were to be performed according to a regular and predictable routine. At the beginning of each working day, the tasks were assigned according to a timetable that included small breaks between the tasks.
iv) A poor level of performance in tasks involving visual-spatial skills, e.g. difficulty in composing jigsaw puzzles or in completing a drawing [1, 4, 8].

We adopted two strategies to address this issue:

1. the use of computers instead of paper and pencil;
2. smaller jigsaw puzzles with a support function for younger children.

Research on Williams Syndrome has also clearly highlighted the presence, in WS patients, of the characteristics listed below, which may be considered as “protective factors” favoring the best possible adaptation to the environment. We decided to take these aspects into account in the definition and design of the Empowerment process.

i) Long-term and physiognomic memory skills. WS individuals’ neurocognitive mechanisms involved in face recognition do not differ from those of individuals with a normal development [16]. This ability may be very important for building a social network.
ii) Great sociability, agreeableness and empathy.
  Based on this aspect, we decided to include, among the various tasks specifically assigned to the patient, the reception of children and their families in the Playroom.
iii) Excellent command of native tongue and a natural flair for foreign languages.
  In order to exploit this characteristic, we often involved our patient in the interaction with the parents of foreign inpatients. She was thus offered the possibility to communicate in a foreign language and perceive herself as an efficient and competent person.
iv) A natural bent for music/musical skills.
  We actively involved her in a music festival organized in the premises of the Hospital.
v) Adequate social interaction skills.
  Our patient in particular showed adequate interaction skills with adults and small children (aged less than 2 years), but poor interaction skills with older children. For this reason, the function of the tutor was to act as a “model” to be imitated in play activities with other children, showing our patient how to start and maintain an adequate interaction with other children.

Based on the above, and considering the specific personality of our WS patient, we devised an activity plan, within the work context of the Hospital, for the first and second semester, and we devided it into steps, having each a specific goal.

Our aim was to make the best use of our patient’s abilities, while at the same time trying to limit her deficit areas, and help her acquire awareness on her own strengths and weaknesses.

Our intervention is not to be considered as a program aiming at “correcting” the lacking aspects of our patient. On the contrary, we wanted the program to have a “transformative-evolutionary” function, through encouraging our patient to use her internal and external resources, so as to foster the growth of her “internal power”.

## Results

The patient ensured continuous attendance and showed a strong determination and commitment. She got along well with the operators and with all those in charge of the different activities, and developed open and friendly relationships. She questioned herself, accepted advice, carefully observed the work of other people, and established an empathetic relationship with her tutor. In both the working contexts where the project was implemented, we observed a gradual improvement of her knowledge, her practical and social skills, and her personal motivation while working.

During the first period of the project, a constant presence of the tutor was necessary to support and help her while performing her tasks. At a later stage, the tutor only monitored the activities. This was possible thanks to her increased autonomy and self-management skills.

## Conclusions

This intervention project was implemented at the Bambino Gesù Children’s Hospital in Rome with the active participation of a patient affected by Williams Syndrome. The aim of the project was to outline specific strategies to improve personal functioning of WS individuals.

As with many other genetic diseases, there is no specific cure for Williams Syndrome. Rehabilitation therapy is necessary to develop better cognitive and social skills and personal self-sufficiency, thus enhancing the patient’s dignity and sense of self-value. Self-identity and professional role are two factors that trigger off, corroborate and complement each other, in a complex circular phenomenon [13].

Considering the intra-individual variability in Williams Syndrome, it is not realistic to believe that all WS patients can find a job. This belief may generate false expectations in both the patients and their families. It is therefore necessary to make sure that these patients can avail themselves of intervention programs meeting their specific needs and encouraging their potential, in order to devise the best possible reintegration program [19].

A first step may be to devise programs that help patients develop skills of personal and social self-sufficiency from a very young age. At a later stage, patients may be supported with gradual and targeted interventions when entering the labor market. Just like anybody else, WS patients wish to form attachments, be perceived as adults, and make their own living.

In light of the above, this project represents a pilot and unique experience in Italy and may serve as a reference model for other national institutions. Furthermore, it can serve as a positive stimulus and a model to encourage healthcare institutions and educational programs to dedicate greater attention to the psycological and social well-being of patients affected by rare conditions.

“There are no normal people and non-normal people, but women and men with strengths and weaknesses and it is up to society to make sure that everyone can feel free, and no one feel alone”

Franco Basaglia

## Abbreviation

WS: Williams Syndrome
